# *Helicobacter pylori* Induced Gastric Immunopathology Is Associated with Distinct Microbiota Changes in the Large Intestines of Long-Term Infected Mongolian Gerbils

**DOI:** 10.1371/journal.pone.0100362

**Published:** 2014-06-18

**Authors:** Markus M. Heimesaat, André Fischer, Rita Plickert, Tobias Wiedemann, Christoph Loddenkemper, Ulf B. Göbel, Stefan Bereswill, Gabriele Rieder

**Affiliations:** 1 Department of Microbiology and Hygiene, Charité - University Medicine Berlin, Berlin, Germany; 2 German Research Center for Environmental Health, Helmholtz Zentrum München, Munich, Germany; 3 Department of Pathology/Research Center ImmunoSciences (RCIS), Charité - University Medicine Berlin, Berlin, Germany; 4 Division of Molecular Biology, Department of Microbiology, University of Salzburg, Salzburg, Austria; Vanderbilt University Medical Center, United States of America

## Abstract

**Background:**

Gastrointestinal (GI) inflammation in mice and men are frequently accompanied by distinct changes of the GI microbiota composition at sites of inflammation. *Helicobacter (H.) pylori* infection results in gastric immunopathology accompanied by colonization of stomachs with bacterial species, which are usually restricted to the lower intestine. Potential microbiota shifts distal to the inflammatory process following long-term *H. pylori* infection, however, have not been studied so far.

**Methodology/Principal Findings:**

For the first time, we investigated microbiota changes along the entire GI tract of Mongolian gerbils after 14 months of infection with *H. pylori* B8 wildtype (WT) or its isogenic Δ*cagY* mutant (MUT) strain which is defective in the type IV secretion system and thus unable to modulate specific host pathways. Comprehensive cultural analyses revealed that severe gastric diseases such as atrophic pangastritis and precancerous transformations were accompanied by elevated luminal loads of *E. coli* and enterococci in the caecum and together with *Bacteroides/Prevotella* spp. in the colon of *H. pylori* WT, but not MUT infected gerbils as compared to naïve animals. Strikingly, molecular analyses revealed that *Akkermansia*, an uncultivable species involved in mucus degradation, was exclusively abundant in large intestines of *H. pylori* WT, but not MUT infected nor naïve gerbils.

**Conclusion/Significance:**

Taken together, long-term infection of Mongolian gerbils with a *H. pylori* WT strain displaying an intact type IV secretion system leads to distinct shifts of the microbiota composition in the distal uninflamed, but not proximal inflamed GI tract. Hence, *H. pylori* induced immunopathogenesis of the stomach, including hypochlorhydria and hypergastrinemia, might trigger large intestinal microbiota changes whereas the exact underlying mechanisms need to be further unraveled.

## Introduction

Chronic *Helicobacter (H.) pylori* infection represents a significant health burden affecting approximately half of the world’s population [1]. Even though the vast majority of infected individuals remains asymptomatic or displays rather minor unspecific symptoms, the pathogen can cause chronic gastritis, peptic ulcer, gastric adenocarcinoma or mucosa-associated lymphoid tissue lymphoma [2], [3]. As a consequence of epidemiological studies, in 1994 the WHO has declared *H. pylori* a class I carcinogen [4]. *H. pylori* produce a plethora of virulence factors inducing gastric immunopathology. Among these, the vacuolating cytotoxin A (VacA) and cytotoxin-associated antigen A (CagA) have been studied intensively [5], [6], [7], [8], [9].

Strains carrying a *cag* pathogenicity island (*cagPAI*) are able to translocate the effector protein CagA via the type IV secretion system (T4SS) into the host cell. CagA gets tyrosine phosphorylated by host kinases and interferes with the signal transduction pathways regulating cell polarity, inflammation, proliferation, and apoptosis [5], [10]. The *H. pylori* mutant Δ*cagY* is defective in the T4SS, thus representing a less virulent strain, which lacks the ability to induce a corpus dominant atrophic gastritis as confirmed in our previous Mongolian gerbil study [9]. As compared to mice, the Mongolian gerbil infection model has been proven to better mimic key features of pathophysiology and long-term sequelae in *H. pylori* infected humans (reviewed in [11], [12], [13], [14]). Following *H. pylori* infection of gerbils, like in humans the gastric inflammatory process starts in the antrum mucosa and further expands to the corpus whereas in infected mice the antrum is rarely involved. Importantly, *H. pylori* type I strains expressing CagA, VacA and a functional type IV secretion system only persist in a Mongolian gerbil, but not murine stomach in a genetically stable fashion. In addition, infected gerbils develop severe gastric lesions such as peptic ulcer and gastric adenocarcinoma [11], [12], [13], [14].

Recent reports have highlighted that *H. pylori* infection is associated with changes in the gastric microenvironment, which in turn affects the gastric microbiota composition. In our own study, for instance, we have shown that following *H. pylori* infection bacterial species usually restricted to the lower intestinal tract (such as *Bacteroides/Prevotella* spp. and clostridia, among others) were present in stomach samples of *H. pylori* infected mice, whereas *Lactobacillus* spp. were predominant in control animals [15]. Furthermore, in a previous report Mongolian gerbils with gastritis and duodenitis harbored significantly higher *Bacteroides* spp. numbers at sites of inflammation 12 weeks following *H. pylori* infection [16].

So far, microbiota changes in *H. pylori* infected Mongolian gerbils have only been investigated at sites of inflammation itself [16], [17], [18]. Therefore, for the first time we investigated potential changes of the microbiota composition distal to the inflammatory process, particularly in long-term *H. pylori* infection. Results represent a comprehensive survey of the microbiota composition within the entire gastrointestinal (GI) tract of Mongolian gerbils 14 months following *H. pylori* wildtype infection as compared to its isogenic Δ*cagY* mutant strain applying cultural and molecular methods.

## Results

### Inflammatory Responses in the Gastrointestinal Tract of Mongolian Gerbils Following Long-term *H. pylori* Infection

**Figure 1:**
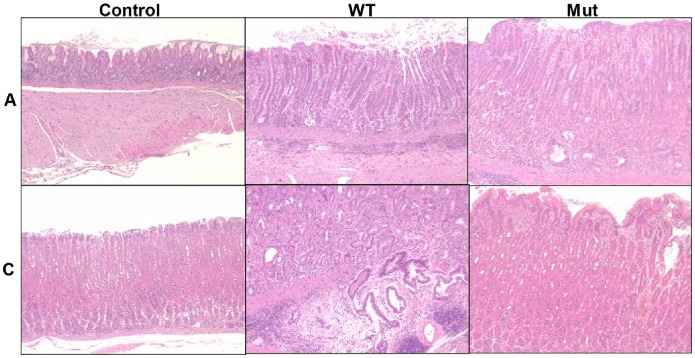
Gastric histopathology in Mongolian gerbils 14 months following *H. pylori* infection. Mongolian gerbils were infected with *H. pylori* wildtype strain B8 (WT; middle panels) or *H. pylori* mutant strain lacking *cagY* (Mut; right panels) as described in Methods. Fourteen months following infection, stomach biopsies separated in antrum (A) and corpus (C) mucosa were taken and paraffin sections stained with H&E. Uninfected age-matched animals served as negative controls (Control; left panels). Representative photomicrographs are shown (100 x magnification).

Acute and chronic GI inflammation is accompanied by shifts within the intestinal microbiota composition at sites of inflammation. In the presented study, we were interested whether long-term *H. pylori* infection results in luminal microbiota changes at sites of gastric inflammation or beyond in the distal GI tract. To address this question Mongolian gerbils were orally infected with *H. pylori* B8 wildtype (WT) or its isogenic B8Δ*cagY* mutant (MUT) strain. Fourteen months thereafter, either *H. pylori* WT and MUT strain could be re-isolated at comparable loads from the stomach in 66.7% and 100% of infected Mongolian gerbils, respectively. In *H. pylori* WT and MUT infected animals, gastric pathology was characterized by lymphoid aggregates in the antrum (100% with either strain) and corpus (100% and 33.3%, respectively), erosions (100% and 83.3%, respectively), ulcers (75.0% and 0%, respectively), parietal cell atrophy (100% and 33.3%, respectively), and metaplastic changes (90.9% and 33.3%, respectively), gastritic cystica profunda (75.0% and 33.3%, respectively), and focal dysplasia (25.0% and 0%, respectively) (**Fig. 1** **,** **Table 1**). After the establishment of a severe pangastritis in *H. pylori* WT strain infected animals, gerbils developed significant increases in pH values from 1.5 to 4 (hypochlorhydria) and plasma gastrin concentrations (hypergastrinemia) (refer to [9]).

**Table 1:** Gastric pathology in Mongolian gerbils.

Animal group	Number ofanimals	Duration of experiment/time of infection (months)	Gastric pathology
Uninfected control	12	14	None
B8 infected (WT)	11	14	Severe inflammation in antrum and corpus mucosa;Lymphoid aggregates in antrum and corpus mucosa;Hyperplasia in antrum and corpus mucosa;Erosions and gastric ulcer; Parietal cell atrophy in corpus mucosa;Mucous gland metaplasia; Gastritis cystica profunda;Focal dysplasia; Gastric pH: 4
B8-Δ*cagY* infected (Mut)	6	14	Moderate and mild inflammation in antrum and corpus mucosa,respectively; Lymphoid aggregates mainly in antrum mucosa;Hyperplasia in antrum mucosa only; Erosions but no gastric ulcer;Rarely observed atrophy, metaplasia, or gastritis cystic profunda;No dysplasia; Gastric pH: 1–2

**Figure 2:**
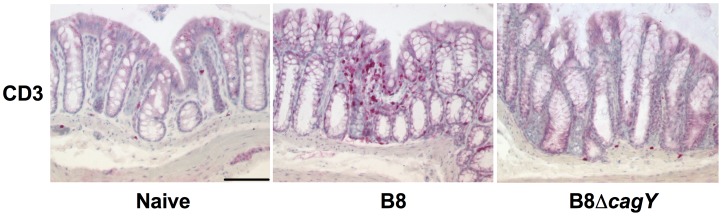
*In situ* T lymphocyte responses in colonic sections of Mongolian gerbils 14 months following *H. pylori* infection. Mongolian gerbils were infected with *H. pylori* wildtype strain B8 (B8; middle) or *H. pylori* mutant strain lacking *cagY* (B8Δ*cagY*; right). Fourteen months following infection, colonic biopsies were taken and paraffin sections stained for CD3 by immunohistochemistry to visualize T lymphocytes *in situ*. Uninfected age-matched animals served as negative controls (Naïve; left). Representative photomicrographs of positively stained cells are shown (200 x magnification, scale bar 50 µm).

Of note, significant histopathological signs of inflammation could not be observed in hematoxylin and eosin (H&E) stained paraffin sections derived from the small and large intestinal tract of infected animals (not shown). To further assess quantitative pro-inflammatory immune cell responses in the colonic mucosa of infected Mongolian gerbils, we performed *in situ* immunohistochemical stainings of colonic paraffin sections with an antibody against CD3 to visualize T lymphocytes. *H. pylori* WT infected gerbils displayed significantly higher numbers of T lymphocytes in their colonic mucosa as compared to MUT infected or naïve animals 14 months post infection (p.i.) **(** **Fig. 2** **)**.

### Distinct Microbiota Changes in the Large, but not small Intestinal Tract of Mongolian Gerbils Following Long-term *H. pylori* Infection

**Figure 3:**
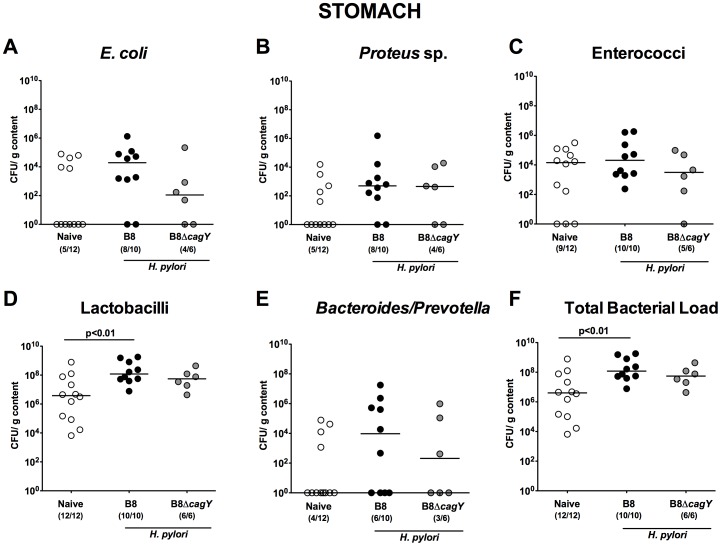
Stomach microbiota composition in Mongolian gerbils 14 months following *H. pylori* infection. Fourteen months following oral infection of Mongolian gerbils with *H. pylori* wildtype strain B8 (B8; black circles) or *H. pylori* mutant strain lacking *cagY* (B8Δ*cagY*; grey circles), the microbiota composition of luminal **stomach** contents were quantitatively analyzed by culture as described in Methods. Uninfected age-matched animals served as negative controls (Naïve; white circles). Numbers of live (**A**) *E. coli*, (**B**) *Proteus* sp., (**C**) Enterococci, (**D**) Lactobacilli, (**E**) *Bacteroides/Prevotella* spp. as well as the (**F**) total bacterial load are indicated as colony forming units (CFU) per g luminal content. Numbers of animals harboring the respective bacterial species are given in parentheses. Medians and significance levels (p*-*values) determined by Mann-Whitney-U test are indicated. Data shown were pooled from three independent experiments.

We next performed a comprehensive cultural survey of the microbiota composition in the entire GI tract of long-term *H. pylori* infected Mongolian gerbils. Fourteen months following *H. pylori* WT or MUT infection, the total bacterial load increased by almost two orders of magnitude in the stomach lumen due to increased numbers of *Lactobacillus* spp. as compared to uninfected controls, but only reached statistical significance in the *H. pylori* WT group (p<0.01) **(** **Fig. 3** **)**. Interestingly, only in 30–40% of naïve gerbils Gram-negative species such as *E. coli*, *Proteus* sp. and *Bacteroides/Prevotella* spp. could be cultured from the gastric lumen (with a median of <10°CFU per g for either bacterial species). During 14 months of long-term infection with the *H. pylori* WT or MUT strain, however, a trend towards higher gastric Gram-negative commensal loads was observed (approximately four and two orders of magnitude, respectively; not significant, n.s.) when compared to uninfected gerbils (**Fig. 3**).

**Figure 4:**
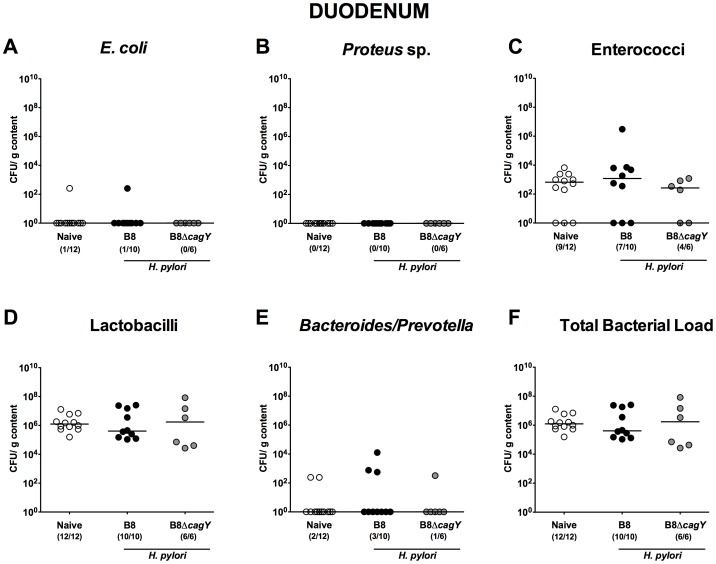
Duodenal microbiota composition in Mongolian gerbils 14 months following *H. pylori* infection. Fourteen months following oral infection of Mongolian gerbils with *H. pylori* wildtype strain B8 (B8; black circles) or *H. pylori* mutant strain lacking *cagY* (B8Δ*cagY*; grey circles), the microbiota composition of luminal **duodenum** contents were quantitatively analyzed by culture as described in Methods. Uninfected age-matched animals served as negative controls (Naïve; white circles). Numbers of live (**A**) *E. coli*, (**B**) *Proteus* sp., (**C**) Enterococci, (**D**) Lactobacilli, (**E**) *Bacteroides/Prevotella* spp. as well as the (**F**) total bacterial load are indicated as colony forming units (CFU) per g luminal content. Numbers of animals harboring the respective bacterial species are given in parentheses and medians indicated. Data shown were pooled from three independent experiments.

**Figure 5:**
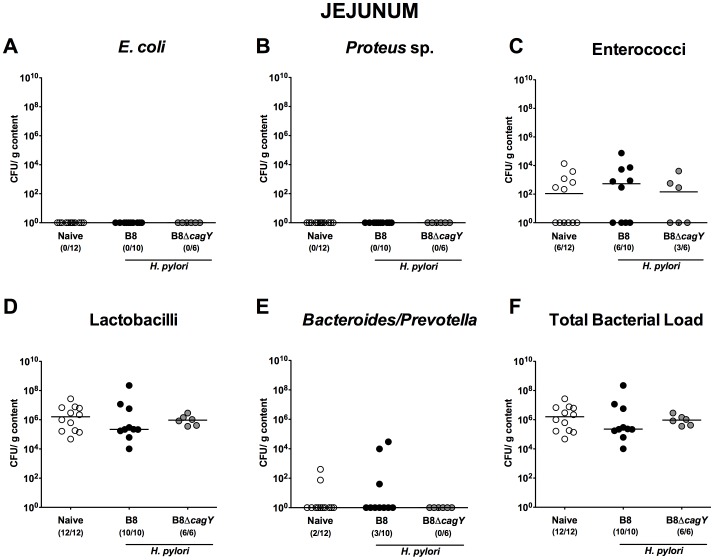
Jejunal microbiota composition in Mongolian gerbils 14 months following *H. pylori* infection. Fourteen months following oral infection of Mongolian gerbils with *H. pylori* wildtype strain B8 (B8; black circles) or *H. pylori* mutant strain lacking *cagY* (B8Δ*cagY*; grey circles), the microbiota composition of luminal **jejunum** contents were quantitatively analyzed by culture as described in Methods. Uninfected age-matched animals served as negative controls (Naïve; white circles). Numbers of live (**A**) *E. coli*, (**B**) *Proteus* sp., (**C**) Enterococci, (**D**) Lactobacilli, (**E**) *Bacteroides/Prevotella* spp. as well as the (**F**) total bacterial load are indicated as colony forming units (CFU) per g luminal content. Numbers of animals harboring the respective bacterial species are given in parentheses and medians indicated. Data shown were pooled from three independent experiments.

**Figure 6:**
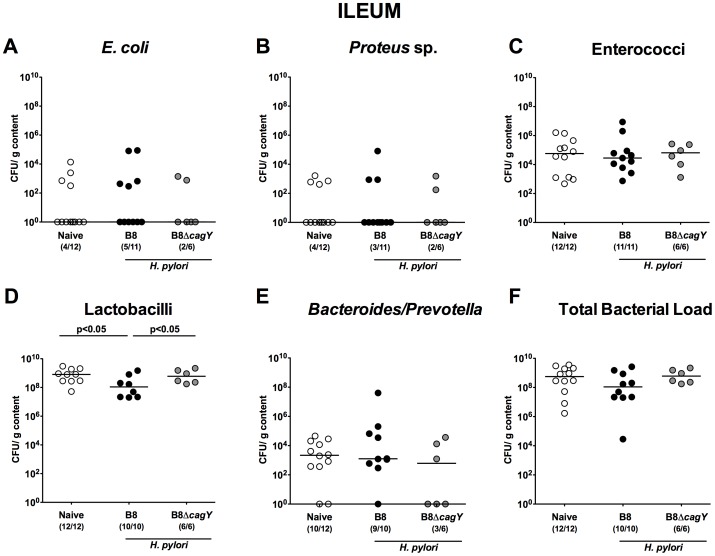
Ileal microbiota composition in Mongolian gerbils 14 months following *H. pylori* infection. Fourteen months following oral infection of Mongolian gerbils with *H. pylori* wildtype strain B8 (B8; black circles) or *H. pylori* mutant strain lacking *cagY* (B8Δ*cagY*; grey circles), the microbiota composition of luminal **ileum** contents were quantitatively analyzed by culture as described in Methods. Uninfected age-matched animals served as negative controls (Naïve; white circles). Numbers of live (**A**) *E. coli*, (**B**) *Proteus* sp., (**C**) Enterococci, (**D**) Lactobacilli, (**E**) *Bacteroides/Prevotella* spp. as well as the (**F)** total bacterial load are indicated as colony forming units (CFU) per g luminal content. Numbers of animals harboring the respective bacterial species are given in parentheses. Medians and significance levels (p*-*values) determined by Mann-Whitney-U test are indicated. Data shown were pooled from three independent experiments.

In proximal parts of the small intestine such as duodenum (**Fig. 4**) and jejunum (**Fig. 5**) no differences in the luminal microbiota composition of infected versus non-infected gerbils could be observed. In the ileal lumen of *H. pylori* WT infected animals, however, lower *Lactobacillus* spp. numbers (approximately 1.5 orders of magnitude; p<0.05) were detected as compared to naïve or with the *H. pylori* MUT strain infected gerbils at 14 months p.i. (**Fig. 6**).

**Figure 7:**
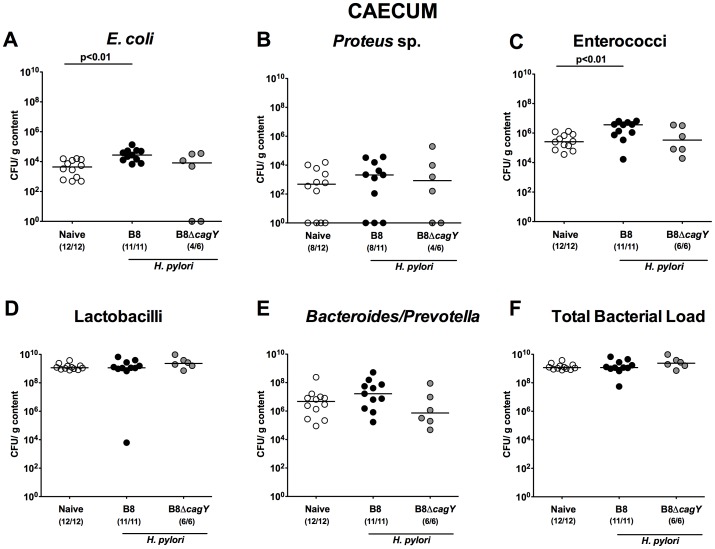
Caecal microbiota composition in Mongolian gerbils 14 months following *H. pylori* infection. Fourteen months following oral infection of Mongolian Gerbils with *H. pylori* wildtype strain B8 (B8; black circles) or *H. pylori* mutant strain lacking *cagY* (B8Δ*cagY*; grey circles), the microbiota composition of luminal **caecum** contents were quantitatively analyzed by culture as described in Methods. Uninfected age-matched animals served as negative controls (Naïve; white circles). Numbers of live (**A**) *E. coli*, (**B**) *Proteus* sp., (**C**) Enterococci, (**D**) Lactobacilli, (**E**) *Bacteroides/Prevotella* spp. as well as the (**F**) total bacterial load are indicated as colony forming units (CFU) per g luminal content. Numbers of animals harboring the respective bacterial species are given in parentheses. Medians and significance levels (p*-*values) determined by Mann-Whitney-U test are indicated. Data shown were pooled from three independent experiments.

**Figure 8:**
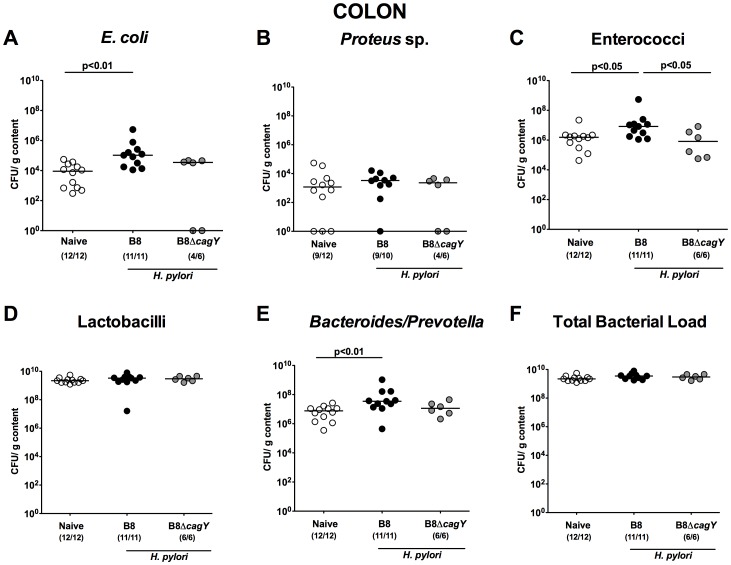
Colonic microbiota composition in Mongolian gerbils 14 months following *H. pylori* infection by culture. Fourteen months following oral infection of Mongolian gerbils with *H. pylori* wildtype strain B8 (B8; black circles) or *H. pylori* mutant strain lacking *cagY* (B8Δ*cagY*; grey circles), the microbiota composition of luminal **colon** contents were quantitatively analyzed by culture as described in Methods. Uninfected age-matched animals served as negative controls (Naïve; white circles). Numbers of live (**A**) *E. coli*, (**B**) *Proteus* sp., (**C**) Enterococci, (**D**) Lactobacilli, (**E**) *Bacteroides/Prevotella* spp. as well as the (**F**) total bacterial load are indicated as colony forming units (CFU) per g luminal content. Numbers of animals harboring the respective bacterial species are given in parentheses. Medians and significance levels (p*-*values) determined by Mann-Whitney-U test are indicated. Data shown were pooled from three independent experiments.

Strikingly, in the distal compartment of the GI tract, such as the caecum (**Fig. 7**) and colon (**Fig. 8**), *H. pylori* WT, but not MUT strain infected gerbils harbored significantly higher *E. coli* and *Enterococcus* spp. loads (1.0–1.5 orders of magnitude; p<0.01) 14 months following infection when compared to naïve animals. In addition, numbers of *Bacteroides/Prevotella* spp. were up to 100 times higher in colonic lumen of *H. pylori* WT infected animals as compared to naïve controls (**Fig. 8E**). Notably, no significant differences in microbiota composition could be observed in Mongolian gerbils suffering from distinct gastric immunopathology 14 months following infection, irrespective whether *H. pylori* WT strain could be re-isolated or not at necropsy (not shown).

**Figure 9:**
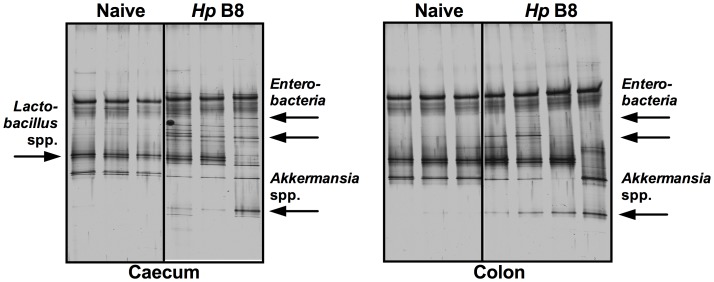
Molecular caecum and colon microbiota analysis in Mongolian gerbils 14 months following *H. pylori* infection. Fourteen months following oral infection of Mongolian gerbils with *H. pylori* wildtype strain B8 (*Hp* B8; right lanes within each panel), the microbiota composition of luminal **caecum** (left panel) and **colon** (right panel) contents were subjected to PCR-DGGE analysis of PCR-amplified total bacterial 16S rRNA gene fragments as described in Methods, and compared to uninfected age-matched controls (Naïve; left lanes within each panel). Sequence analysis revealed that indicated DNA bands appearing 14 months following *H. pylori* B8 infection refer to enterobacteria and *Akkermansia* spp. Shown DGGE profiles of the bacterial microbiota are representative for three independent experiments.

We finally applied molecular methods for detection of fastidious or uncultivable bacterial species in the entire GI tract. Genetic fingerprints generated by PCR-based denaturing gradient gel electrophoresis (DGGE) and subsequent sequence analyses revealed that most prominent bacterial DNA bands occurring in caecal and colonic specimens 14 months following *H. pylori* WT, but not MUT strain infection referred to enterobacteria (**Fig. 9**). Thereby the increased abundance of commensal *E. coli* upon long-term *H. pylori* WT infection as detected by culture could be independently confirmed. Strikingly, *Akkermansia*, an uncultivable species involved in mucus degradation, could exclusively be detected in the caecal and colonic lumen of long-term *H. pylori* WT, but neither MUT infected (not shown) nor naïve gerbils (**Fig. 9**). Interestingly, genetic fingerprints from luminal samples taken from either stomach, duodenum, jejunum or ileum were comparable in *H. pylori* WT, MUT strain infected and naïve gerbils, respectively (not shown), further supporting results derived from culture. Taken together, long-term *H. pylori* WT infection of Mongolian gerbils leads to distinct quantitative as well as qualitative changes of the microbiota composition within the uninflamed distal, but not proximal inflamed GI tract.

## Discussion

In the study presented here we performed a comprehensive survey of microbiota changes along the entire GI tract in Mongolian gerbils following long-term infection with *H. pylori* for 14 months comparing B8 wildtype with its isogenic *cagY* mutant strain defective in the type IV secretion system. Most notably, we for the first time extended the microbiota analyses to locations distal “classical” sites of inflammation (such as stomach and duodenum) to the lower small and large intestines. Our presented data are well in line with our studies in other rodent inflammation models revealing that acute and chronic inflammation of the small as well as large intestinal tract in mice is associated with distinct shifts of the microbiota composition towards overgrowth with commensal *E. coli*, *Bacteroides/Prevotella* spp. and enterococci at sites of inflammation [19], [20], [21], [22], [23]. Given that in the presented study the significant microbiota shifts were exclusively detected following infection with the *H. pylori* WT strain able to translocate the effector protein CagA into the host cells, it is somewhat surprising, though, that at site of gastric inflammation no statistical significant differences in microbial colonization densities could be observed. As we could show in previous studies [9], [10], *H. pylori* induced gastritis in antrum mucosa of Mongolian gerbils is *cagPAI*-independent, whereas a severe inflammation of the corpus mucosa is induced via a *cagPAI*-dependent mechanism. Only *H. pylori* WT infected gerbils developed a pangastritis with a complete atrophy of the parietal cells resulting in a significant up-rise of the intragastral pH value (hypochlorhydria) followed by hypergastrinemia. Moreover, CagA translocation and hypochlorhydria have been shown to be pivotal prerequisites for *H. pylori* induced long-term sequelae in humans such as atrophic gastritis, dysplasia or gastric adenocarcinoma [24], [25], [26], [27]. Furthermore, most of these animals also developed a mucous gland metaplasia producing atypical mucus. This is in sharp contrast to *H. pylori* MUT strain infected gerbils that do not develop such sequelae. One must take into account, however, that the observed lack of statistical significant differences in gastric bacterial colonization densities might be due to the relatively high standard deviations in either group since there was a trend towards four orders of magnitude higher (median) *E. coli* and *Bacteroides/Prevotella* spp. loads in *H. pylori* WT, but not MUT strain infected gerbils at 14 months p.i. when compared to naïve animals.

In a previous molecular study of the gastric microbiota composition we could demonstrate that *E. coli* and *Bacteroides/Prevotella* spp. were exclusively abundant in stomachs of mice 8 weeks following *H. pylori* infection, but absent in uninfected controls [15]. A possible explanation for the gastric abundance of commensals that are usually restricted to the intestines could be that following chronic *H. pylori* infection the “acidic barrier” breaks (and thus the pH within the stomach lumen increases). Subsequently Gram-negative species such as *E. coli* and *Bacteroides/Prevotella* spp. perorally ingested by the coprophagous rodents via their own feces have an advantage to grow in the formerly hostile environment and win the intraluminal competition with other commensal bacteria for niches and nutrients in the stomach. In contrast to mice, however, Mongolian gerbils tend not to coprophage their own feces if supplied with excess food. Therefore, gerbils kept under SPF conditions will not ingest intestinal commensals. This would explain the lack of significant abundance of Gram-negative intestinal commensals in the stomach of gerbils here.

In the past some studies attempted to profile the microbiota of *H. pylori*-infected Mongolian gerbils at sites of inflammation [16], [17], [18], [28]. Yin et al. previously showed that gerbils in which the pathogen could still be re-isolated 12 weeks following *H. pylori* infection harbored higher *Bacteroides, Enterococcus and Staphylococcus* spp., but tended to harbor lower lactobacilli loads in their inflamed stomach and duodenum as compared to animals having expelled the pathogen in the meantime [16]. Furthermore, the gastric and duodenal microbiota composition in *H. pylori*-negative gerbils following infection appeared to be similar to naïve, uninfected animals. The authors concluded that the abundant species had adapted to the *H. pylori* induced changes of the intraluminal milieu at site of infection and inflammation, whereas the lactobacilli population had not [16]. Whether the lower lactobacilli densities was a consequence of *H. pylori* infection (due to milieu changes and lack of adaptation) or had initially rather facilitated the pathogen’s establishment in the stomach cannot be answered. In contrast, in our study 14 months following *H. pylori* infection, gerbils exhibited even significantly (two orders of magnitude) higher lactobacilli and total bacterial loads in the stomach as compared to uninfected animals most likely as a consequence of the hypochlorhydria and, hence, increased pH within the gastric lumen. In addition, a different repertoire of expressed β-defensins [29] and/or abundance of B lymphocyte subsets [30] in lymphoid aggregates of *H. pylori*-infected as compared to naïve gerbils might have contributed to the observed differences in gastric lactoballi loads. Whereas in our study gerbils had been infected for over one year, Yin et al. had performed their analyses 12 weeks p.i. Of note, the microbiota changes in the interplay with pathogen and host responses are a dynamic system and substantially change over time. Given that here we have not assessed the commensal microbiota composition to an earlier time point, results derived following short-term *H. pylori* infection might differ for several (patho-) physiological reasons.

In our study, applied molecular methods (such as PCR-based DGGE analysis with subsequent sequencing of DNA bands of interest) not only confirmed culture-based results (e.g. enterobacterial such as *E. coli* overgrowth). Strikingly, *Akkermansia*, an un-cultivated species involved in mucus degradation [31], could be detected in the caecum and colon of *H. pylori* WT, but not MUT strain infected or naïve gerbils. This suggests that the accumulation of *Akkermansia* is driven by *H. pylori* induced inflammation and, hence, depends on a functional Cag pathogenicity island. Thus, intestinal abundance of bacterial species further compromising host barrier function by mucus degradation, might be one important puzzle piece in the complex pathogenesis of *H. pylori*-induced sequelae, also in humans.

Overall, it is most surprising, however, that in our study even though the *H. pylori* induced immunopathology was prominent within the stomach, the microbiota shifts became exclusively overt in the large and not small intestinal tract where no mucosal histopathological changes of the intestinal mucosa could be observed. Interestingly, we were able to detect increased numbers of CD3+ cells in the large (but not small) intestinal mucosa and lamina propria in long-term *H. plyori*-WT, but not MUT strain infected Mongolian gerbils. It is tempting to speculate that recruited T lymphocytes might subsequently induce the release of cytokines and chemokines, which in turn promote further inflammatory responses leading to a compromised epithelial barrier function. As a consequence, a broken epithelial barrier might lead to changes within the intraluminal milieu subsequently promoting the luminal growth of intestinal commensals such as Gram-negative species as we could show in murine acute and chronic inflammation of the small as well as large intestinal tract [19], [20], [21], [22], [23]. In addition, one might speculate that via the neuroendocrine route, for instance, mediators (e.g. gastrin, somatostatin, cytokines, low proton concentration, among other factors) released in the stomach during *H. pylori* infection and induced immunopathology [32], [33] exert their effect in the lower intestinal tract. Of note, patients suffering from *H. pylori* infection and hypochlorhydria occasionally complain about rather unspecific intestinal symptoms such as irregular bowel movements, flatulence or abdominal pain of otherwise unknown origin [34], [35], which might be attributed to bacterial microbiota changes (e.g. overgrowth of some commensals and disappearance of others from the complex bacterial community) in the lower intestine during the disease process.

Taken together, long-term infection of Mongolian gerbils with an *H. pylori* WT strain displaying an intact type IV secretion system leads to distinct shifts of the microbiota composition in the distal uninflamed, but not proximal inflamed GI tract. Hence, *H. pylori* induced immunopathogenesis of the stomach, including hypochlorhydria and hypergastrinemia, might trigger large intestinal microbiota changes whereas the exact underlying mechanisms need to be further unraveled.

## Materials and Methods

### Ethics Statement

All animal experiments were conducted according to the European Guidelines for animal welfare (2010/63/EU) with approval of the commission for animal experiments headed by the “Regierung von Oberbayern” (AZ 55.2-1-54-2531-41/04 and 55.2-1-54-2531-78/05). Animal welfare was monitored twice daily by assessment of clinical conditions.

### Bacterial Strains

*Helicobacter pylori* B8 (WT), a recently sequenced ([36]; B128 parental strain), Mongolian gerbil-adapted type I-strain (CagA, VacA: s1m2) and its isogenic mutant B8Δ*cagY* (MUT) were used in this study as previously described [10]. Both strains carried a chromosomal streptomycin resistance cassette allowing quantitative recovery of *H. pylori* from the gerbil stomach by antibiotic selection (streptomycin 250 mg/L; [37]) as described earlier [9], [10].

### Animals and Infection Experiments

Outbred Mongolian gerbils from our own breeding colony (Max von Pettenkofer-Institute, LMU Munich, Germany) were bred and maintained under specific pathogen free (SPF) conditions and housed in SEALSAFE IVC cages (H-Temp, Tecniplast, Hohenpeissenberg, Germany) in an air-conditioned biohazard room (room temperature, 23±5%; 12/12 hours light/dark cycle) with free access to a commercial gerbil diet (ssniff Gerbil, SSNIFF, Soest, Germany) and sterile tap water. Female animals at the age of 8–12 weeks were challenged orogastrically three times over five consecutive days with approximately 10^9^ viable *H. pylori* B8 wildtype or *H. pylori* B8Δ*cagY* mutant strain. Age-matched control animals were incubated with identical volumes of sterile Brucella broth alone. Animals were sacrificed after defined time of infection (14 months) by isofluran treatment (Abbott, Germany). Gastrointestinal samples from each Mongolian gerbil were collected in parallel for immunohistological, microbiological, and molecular analyses. The stomach was opened along the great curvature, and the gastric tissue conserved separately in antrum and corpus as previously described [10]. In addition luminal contents of stomach, duodenum, jejunum, ileum, caecum, and colon were collected under sterile conditions in sterile PBS and immediately stored on ice.

### Histopathology and Immunohistochemistry of Gastrointestinal Tissues

Histopathological changes were determined in GI tissue samples immediately fixed in 5% formalin and embedded in paraffin. Sections (5 µm) were stained with H&E and examined by light microscopy (100 x and 400 x magnification). *In situ* immunohistochemical analysis of paraffin sections taken from the colon was performed as described previously [38]. A primary antibody against CD3 (#N1580, Dako, Denmark, dilution 1∶10) was used to visualize T lymphocytes in the colonic mucosa *in situ* (200 x magnification).

### Cultural Analysis of the Luminal Gastrointestinal Mícrobiota

Cultural analysis of the intestinal microbiota along the entire GI tract was performed as described previously [20]. Luminal contents taken from stomach, duodenum, jejunum, ileum, caecum, and distal colon were resuspended in PBS, weighted, and 100 µl aliquots of serial dilutions plated onto solid media (Oxoid). Bacteria were grown at 37°C for 2 days under aerobic or for 4 days under anaerobic conditions, and total numbers determined by colony counting on Columbia blood agar. Bile esculin, McConkey, anaerobe 5% sheep blood agar supplemented with kanamycin and vancomycin, and Rogosa (Merck) media were used for quantitative identification of enterococci, enterobacteria (such as *E. coli* and *Proteus* spp.), *Bacteroides/Prevotella* spp., and lactic acid bacteria (mainly lactobacilli), respectively. Bacteria were subcultivated and further investigated by Gram-staining and by biochemical analysis with the API20E, API50 CH, and API Rapid ID 32A systems (Biomérieux) and confirmed by sequence analyses in cases of inconclusive identification. Results were expressed as colony forming units (CFU) per g of luminal content. The detection limit of viable pathogens was ≈25 CFU per g.

### Molecular Microbiota Analysis

Molecular detection of fastidious or uncultivable bacterial communities in stomach, duodenum, jejunum, ileum, caecum, and distal colon was performed as previously described [20]. Respective luminal contents were removed, resuspended in PBS, and centrifuged (16,000 g/10 min/4°C). The sediment was resuspended in 0.5 ml of lysis buffer (500 mM Tris (pH 9.0), 20 mM EDTA, 10 mM NaCl, 1% SDS) and incubated with proteinase K (2 mg/ml; Sigma-Aldrich) for 1 h at 56°C. After bead beating with zirconium-silica beads (0.2 mm), the total DNA isolated by phenol-chloroform extraction served as template for PCR amplification.

Genetic fingerprints were generated by PCR denaturing gradient gel electrophoresis (PCR-DGGE; [39]. Variable regions 6–8 in bacterial 16S rRNA genes were amplified (denaturation at 95°C/5 min, then 25 cycles of 93°C/45 s, 64°C/1 min, 72°C/1 min, final elongation at 72°C/7 min) from total gut content DNA with GC clamp (underlined) primer GC968F (5′-CGCCCGGGGCGCGCCCCGGGCGGGGCGGGGGCACGGGGGGAACGCGAAGAACCTTAC-3′, nt 968-84 in *E. coli* 16S rRNA) and primer R1378 (5′-CGGTGTGTACAAGGCCCGGGAACG-3′, nt 1401-1378 in *E. coli* 16S rRNA). Amplicons (300 ng) were electrophoresed on a DCode System (Bio-Rad) at 80 V/60°C for 16 h in a polyacrylamide gel containing 35–60% urea/formamide. DNA band profiles were visualized by silver staining. For sequence analysis, DGGE bands were stained with SYBR green I (Fluka), visualized under UV light, and cut off the gel matrix. DNA was eluted by shaking in double-distilled water (ddH2O) overnight at 37°C. After reamplification by PCR, the amplicons were cloned (pCR2.1 TOPO-TA, Invitrogen life technologies) and sequenced for phylogenetic identification.

### Statistical Analysis

Medians and levels of significance were determined using Mann-Whitney-U Test. Two-sided probability (*P*) values ≤0.05 were considered significant. Experiments were repeated twice.

## References

[1] Dooley CP, Cohen H, Fitzgibbons PL, Bauer M, Appleman MD, et al (1989) Prevalence of *Helicobacter pylori* infection and histologic gastritis in asymptomatic persons. N Engl J Med 321: 1562–1566. 2586553 10.1056/NEJM198912073212302

[2] Kuipers EJ, Uyterlinde AM, Pena AS, Roosendaal R, Pals G, et al (1995) Long-term sequelae of *Helicobacter pylori* gastritis. Lancet 345: 1525–1528. 7791437 10.1016/s0140-6736(95)91084-0

[3] Suerbaum S, Michetti P (2002) *Helicobacter pylori* infection. N Engl J Med 347: 1175–1186. 12374879 10.1056/NEJMra020542

[4] International Agency for Research on Cancer (1994) Schistosomes, liver flukes and *Helicobacter pylori*. IARC Working Group on the Evaluation of Carcinogenic Risks to Humans. Lyon, 7–14 June 1994. IARC Monogr Eval Carcinog Risks Hum 61: 1–241. 7715068 PMC7681621

[5] Wessler S, Backert S (2008) Molecular mechanisms of epithelial-barrier disruption by *Helicobacter pylori* . Trends Microbiol 16: 397–405. 18619844 10.1016/j.tim.2008.05.005

[6] Poppe M, Feller SM, Romer G, Wessler S (2007) Phosphorylation of *Helicobacter pylori* CagA by c-Abl leads to cell motility. Oncogene 26: 3462–3472. 17160020 10.1038/sj.onc.1210139

[7] Sewald X, Gebert-Vogl B, Prassl S, Barwig I, Weiss E, et al (2008) Integrin subunit CD18 Is the T-lymphocyte receptor for the *Helicobacter pylori* vacuolating cytotoxin. Cell Host Microbe 3: 20–29. 18191791 10.1016/j.chom.2007.11.003

[8] Hatakeyama M (2006) The role of *Helicobacter pylori* CagA in gastric carcinogenesis. Int J Hematol 84: 301–308. 17118755 10.1532/IJH97.06166

[9] Wiedemann T, Loell E, Mueller S, Stoeckelhuber M, Stolte M, et al (2009) *Helicobacter pylori* cag-Pathogenicity island-dependent early immunological response triggers later precancerous gastric changes in Mongolian gerbils. PLoS One 4: e4754. 19270747 10.1371/journal.pone.0004754 PMC2650263

[10] Rieder G, Merchant JL, Haas R (2005) *Helicobacter pylori* cag-type IV secretion system facilitates corpus colonization to induce precancerous conditions in Mongolian gerbils. Gastroenterology 128: 1229–1242. 15887107 10.1053/j.gastro.2005.02.064

[11] Hirayama F, Takagi S, Yokoyama Y, Iwao E, Ikeda Y (1996) Establishment of gastric *Helicobacter pylori* infection in Mongolian gerbils. J Gastroenterol 31 Suppl 9: 24–28. 8959513

[12] Hirayama F, Takagi S, Kusuhara H, Iwao E, Yokoyama Y, et al (1996) Induction of gastric ulcer and intestinal metaplasia in mongolian gerbils infected with *Helicobacter pylori* . J Gastroenterol 31: 755–757. 8887049 10.1007/BF02347631

[13] Tsukamoto T, Toyoda T, Mizoshita T, Tatematsu M (2013) *Helicobacter pylori* infection and gastric carcinogenesis in rodent models. Semin Immunopathol 35: 177–190. 23111700 10.1007/s00281-012-0357-1

[14] Zhang S, Moss SF (2012) Rodent models of *Helicobacter* infection, inflammation, and disease. Methods Mol Biol 921: 89–98. 23015495 10.1007/978-1-62703-005-2_12 PMC3641907

[15] Aebischer T, Fischer A, Walduck A, Schlotelburg C, Lindig M, et al (2006) Vaccination prevents *Helicobacter pylori*-induced alterations of the gastric flora in mice. FEMS Immunol Med Microbiol 46: 221–229. 16487303 10.1111/j.1574-695X.2005.00024.x

[16] Yin YN, Wang CL, Liu XW, Cui Y, Xie N, et al (2011) Gastric and duodenum microflora analysis after long-term *Helicobacter pylori* infection in Mongolian Gerbils. Helicobacter 16: 389–397. 21923685 10.1111/j.1523-5378.2011.00862.x

[17] Osaki T, Matsuki T, Asahara T, Zaman C, Hanawa T, et al (2012) Comparative analysis of gastric bacterial microbiota in Mongolian gerbils after long-term infection with *Helicobacter pylori* . Microb Pathog 53: 12–18. 22783557 10.1016/j.micpath.2012.03.008

[18] Zaman C, Osaki T, Hanawa T, Yonezawa H, Kurata S, et al (2010) Analysis of the microflora in the stomach of Mongolian gerbils infected with *Helicobacter pylori* . J Gastroenterol Hepatol 25 Suppl 1: S11–14. 20586850 10.1111/j.1440-1746.2009.06215.x

[19] Haag LM, Fischer A, Otto B, Plickert R, Kuhl AA, et al (2012) Intestinal microbiota shifts towards elevated commensal *Escherichia coli* loads abrogate colonization resistance against *Campylobacter jejuni* in mice. PLoS One 7: e35988. 22563475 10.1371/journal.pone.0035988 PMC3341396

[20] Heimesaat MM, Bereswill S, Fischer A, Fuchs D, Struck D, et al (2006) Gram-negative bacteria aggravate murine small intestinal Th1-type immunopathology following oral infection with *Toxoplasma gondii* . J Immunol 177: 8785–8795. 17142781 10.4049/jimmunol.177.12.8785

[21] Heimesaat MM, Fischer A, Jahn HK, Niebergall J, Freudenberg M, et al (2007) Exacerbation of murine ileitis by Toll-like receptor 4 mediated sensing of lipopolysaccharide from commensal *Escherichia coli* . Gut 56: 941–948. 17255219 10.1136/gut.2006.104497 PMC1994376

[22] Heimesaat MM, Fischer A, Siegmund B, Kupz A, Niebergall J, et al (2007) Shift towards pro-inflammatory intestinal bacteria aggravates acute murine colitis via Toll-like receptors 2 and 4. PLoS One 2: e662. 17653282 10.1371/journal.pone.0000662 PMC1914380

[23] Erridge C, Duncan SH, Bereswill S, Heimesaat MM (2010) The induction of colitis and ileitis in mice is associated with marked increases in intestinal concentrations of stimulants of TLRs 2, 4, and 5. PLoS One 5: e9125. 20161736 10.1371/journal.pone.0009125 PMC2817728

[24] Saha A, Hammond CE, Beeson C, Peek RM Jr, Smolka AJ (2010) *Helicobacter pylori* represses proton pump expression and inhibits acid secretion in human gastric mucosa. Gut 59: 874–881. 20581234 10.1136/gut.2009.194795 PMC2980826

[25] Saha A, Backert S, Hammond CE, Gooz M, Smolka AJ (2010) *Helicobacter pylori* CagL activates ADAM17 to induce repression of the gastric H, K-ATPase alpha subunit. Gastroenterology 139: 239–248. 20303353 10.1053/j.gastro.2010.03.036 PMC2902712

[26] Smolka AJ, Backert S (2012) How *Helicobacter pylori* infection controls gastric acid secretion. J Gastroenterol 47: 609–618. 22565637 10.1007/s00535-012-0592-1

[27] Gaddy JA, Radin JN, Loh JT, Zhang F, Washington MK, et al (2013) High dietary salt intake exacerbates *Helicobacter pylori*-induced gastric carcinogenesis. Infect Immun 81: 2258–2267. 23569116 10.1128/IAI.01271-12 PMC3676043

[28] Sun YQ, Monstein HJ, Nilsson LE, Petersson F, Borch K (2003) Profiling and identification of eubacteria in the stomach of Mongolian gerbils with and without *Helicobacter pylori* infection. Helicobacter 8: 149–157. 12662383 10.1046/j.1523-5378.2003.00136.x

[29] Jiang W, Ghosh SK, Flyckt R, Kalinowska M, Starks D, et al (2012) Bacterial colonization and beta defensins in the female genital tract in HIV infection. Curr HIV Res 10: 504–512. 22716110 10.2174/157016212802429848 PMC3427638

[30] Valeur N, Engel P, Carbajal N, Connolly E, Ladefoged K (2004) Colonization and immunomodulation by *Lactobacillus reuteri* ATCC 55730 in the human gastrointestinal tract. Appl Environ Microbiol 70: 1176–1181. 14766603 10.1128/AEM.70.2.1176-1181.2004 PMC348788

[31] Derrien M, Vaughan EE, Plugge CM, de Vos WM (2004) *Akkermansia muciniphila* gen. nov., sp. nov., a human intestinal mucin-degrading bacterium. Int J Syst Evol Microbiol 54: 1469–1476. 15388697 10.1099/ijs.0.02873-0

[32] Selgrad M, Bornschein J, Rokkas T, Malfertheiner P (2012) *Helicobacter pylori*: gastric cancer and extragastric intestinal malignancies. Helicobacter 17 Suppl 1: 30–35. 10.1111/j.1523-5378.2012.00980.x 22958153

[33] Chu S, Schubert ML (2013) Gastric secretion. Curr Opin Gastroenterol 29: 636–641. 24100729 10.1097/MOG.0b013e328365efc7

[34] Huseby ES, White J, Crawford F, Vass T, Becker D, et al (2005) How the T cell repertoire becomes peptide and MHC specific. Cell 122: 247–260. 16051149 10.1016/j.cell.2005.05.013

[35] Yakoob J, Abbas Z, Naz S, Islam M, Jafri W (2012) Virulence markers of *Helicobacter pylori* in patients with diarrhoea-dominant irritable bowel syndrome. Br J Biomed Sci 69: 6–10. 22558797 10.1080/09674845.2012.11669914

[36] Farnbacher M, Jahns T, Willrodt D, Daniel R, Haas R, et al (2010) Sequencing, annotation, and comparative genome analysis of the gerbil-adapted *Helicobacter pylori* strain B8. BMC Genomics 11: 335. 20507619 10.1186/1471-2164-11-335 PMC3091624

[37] Kavermann H, Burns BP, Angermuller K, Odenbreit S, Fischer W, et al (2003) Identification and characterization of *Helicobacter pylori* genes essential for gastric colonization. J Exp Med 197: 813–822. 12668646 10.1084/jem.20021531 PMC2193887

[38] Heimesaat MM, Nogai A, Bereswill S, Plickert R, Fischer A, et al (2010) MyD88/TLR9 mediated immunopathology and gut microbiota dynamics in a novel murine model of intestinal graft-versus-host disease. Gut 59: 1079–1087. 20639251 10.1136/gut.2009.197434

[39] Muyzer G, Smalla K (1998) Application of denaturing gradient gel electrophoresis (DGGE) and temperature gradient gel electrophoresis (TGGE) in microbial ecology. Antonie Van Leeuwenhoek 73: 127–141. 9602286 10.1023/a:1000669317571

